# Clinical features and prognostic factors of salivary adenoid cystic and mucoepidermoid carcinomas

**DOI:** 10.1097/MD.0000000000043409

**Published:** 2025-07-18

**Authors:** Xiaoqin Yang, Ye Meng, Can Xiao, Liya Wang

**Affiliations:** a School of Basic Medical Sciences, Suzhou Medical College of Soochow University, Suchoow, China; b Department of Stomatology, The First Affiliated Hospital of Soochow University, Suchoow, China.

**Keywords:** adenoid cystic carcinoma, mucoepidermoid carcinoma, salivary gland, SEER database, survival

## Abstract

Adenoid cystic carcinoma (ACC) and mucoepidermoid carcinoma (MEC) are common malignant tumors of the salivary glands. This study aimed to compare ACC and MEC regarding clinical features and survival factors. Data from all ACC and MEC cases between 2000 and 2020 were extracted from the US National Cancer Institute’s Surveillance, Epidemiology, and End Results (SEER) database. Clinical features, overall survival (OS), and disease-specific survival (DSS) were assessed for both cancer types. The study included 1426 ACC cases and 3190 MEC cases. In ACC, 580 patients (40.7%) were male, while in MEC, 1494 patients (46.8%) were male. The mean OS was significantly higher for MEC than for ACC (79.33 ± 58.62 months vs 73.44 ± 57.18 months, *P* < .001), and the mean DSS was greater for MEC (46.80 ± 39.26 months vs 29.06 ± 29.79 months, *P* < .001). Independent predictors of worse OS and DSS in ACC included age ≥ 60 years, male gender, submandibular gland involvement, advanced stage, lymph node involvement, distant metastasis, and surgical treatment (all *P* < .05). This study found that MEC has a significantly better prognosis than ACC, with several common factors linked to worse outcomes for both types, including age ≥60 years and male gender, while surgery acted as a significant protective factor.

## 1. Introduction

Adenoid cystic carcinoma (ACC) is a relatively rare tumor that accounts for approximately 3% to 5% of all head and neck malignancies^[1]^ and around 10% of all salivary gland tumors.^[2]^ ACC is characterized by slow growth but an aggressive clinical course, often resulting in late recurrences and distant metastases. The current main treatment strategy for ACC involves surgical resection with negative margins, often followed by adjuvant radiotherapy.^[3]^ However, achieving negative surgical margins can be challenging due to the tumor’s tendency to occur near critical structures and its propensity for perineural invasion (PNI) and intracranial extension, complicating both resection and management.^[4]^ Despite treatment, ACC has a high recurrence rate and a relatively poor long-term prognosis, making the optimization of treatment strategies vital for improving outcomes.^[5]^

Mucoepidermoid carcinoma (MEC), on the other hand, is the most common malignant salivary gland tumor, accounting for 10% to 15% of all salivary gland tumors and 30% of all salivary gland malignancies.^[6,7]^ MEC is comprised of varying proportions of mucous, intermediate, and epidermoid cells, with its prognosis closely linked to tumor grade. Unlike ACC, MEC is more thoroughly studied, with well-established treatment guidelines primarily involving surgical resection, sometimes supplemented with radiotherapy in high-risk cases.^[8]^ MEC generally has a more favorable prognosis, particularly in low-grade cases, although high-grade MECs can exhibit aggressive behavior and have an increased risk of recurrence and metastasis.^[9]^

ACC and MEC, although distinct in terms of histology and behavior, share similarities, particularly their occurrence in the salivary glands and potential for local invasion and metastasis. Comparing these 2 entities is clinically significant as it helps in understanding the differential prognosis, risk factors, and response to treatment modalities. Some studies have explored the clinical course of both malignancies, highlighting notable differences in survival outcomes.^[10–12]^ These comparisons provide a broader context for identifying gaps in current knowledge and guiding the management of these tumors.

This study aimed to comprehensively compare the clinical characteristics and survival factors of ACC and MEC of the salivary glands, using data from the SEER database, to provide valuable insights for enhancing treatment approaches.

## 2. Materials and methods

### 2.1. Data sources

All cases of ACC and MEC of the salivary glands diagnosed between 2000 and 2020 were extracted from the Surveillance, Epidemiology, and End Results (SEER) database, which includes data from 17 registry studies (November 2022 submission, covering 2000–2020). Since SEER is a publicly accessible database with anonymized patient information, ethical approval or informed consent was not required for this study.

### 2.2. Population and data collection

This study included histopathologically confirmed cases of ACC (ICD-O-3 8200) and mucoepidermoid carcinoma (MEC, ICD-O-3 8430), classified according to the WHO Classification of Tumors, 5th edition. Patients with incomplete data were excluded. Clinicopathological information was collected from the SEER database, including age, sex, race, year of diagnosis, primary tumor site, TNM stage, and treatment modalities (surgery, chemotherapy, radiotherapy), as well as survival time. Analyses were conducted using TNM staging from the American Joint Committee on Cancer 6th edition (2004). The endpoints analyzed were overall survival (OS), defined as the time from diagnosis to death from any cause, and disease-specific survival (DSS), defined as the time from diagnosis to death specifically due to the studied disease.

### 2.3. Statistical methods

Data extraction was performed using SEER*Stat software version 8.4.2 (http://seer.cancer.gov). Statistical analyses were conducted using R version 4.3.2. Descriptive statistics were reported as frequencies and percentages. Differences in clinicopathological characteristics between the ACC and MEC groups were analyzed using the chi-square test. Kaplan–Meier survival curves and log-rank tests were used to compare survival outcomes between the groups. Univariate and multivariate Cox regression analyses were performed to identify factors influencing mortality for ACC and MEC. A *P*-value of <.05 was considered statistically significant.

## 3. Results

**Figure 1.**
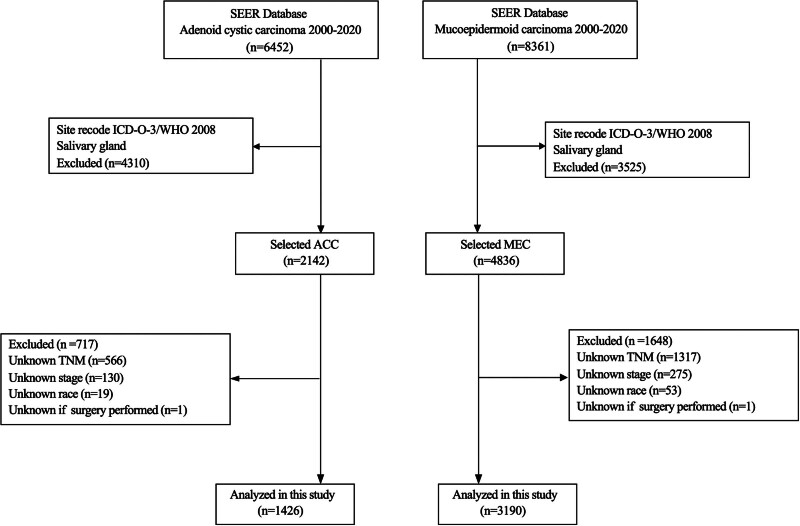
Flowchart depicting the enrollment of patients in this study.

Initially, 6452 cases of ACC and 8361 cases of MEC were identified. After applying the inclusion criteria based on the site recode for salivary gland tumors (ICD-O-3/WHO 2008), 2142 ACC and 4836 MEC cases were retained, excluding 4310 ACC and 3525 MEC cases that did not meet the site criteria. Further exclusions were made due to missing information: 717 ACC cases were excluded for missing TNM staging (n = 566), missing overall stage (n = 130), unknown race (n = 19), and unknown surgical status (n = 1), resulting in 1426 ACC cases for analysis. For MEC, 1648 cases were excluded for missing TNM staging (n = 1317), missing overall stage (n = 275), unknown race (n = 53), and unknown surgical status (n = 1), resulting in 3190 MEC cases for analysis (Fig. 1).

The demographic and clinical characteristics of the patients are summarized in Table 1. Among patients with ACC, the majority were diagnosed between the ages of 50 and 59 years (22.4%, 320 patients), while MEC cases were most frequently diagnosed between the ages of 60 and 69 years (20.3%, 647 patients, *P* < .001). A higher proportion of ACC patients were female compared to MEC patients (59.3% vs 53.2%, *P* < .001). The parotid gland was the primary site of involvement in 46.7% of ACC cases and 86.5% of MEC cases (*P* < .001). ACC cases also demonstrated a higher frequency of stage IV disease (31.2% vs 20.0%), whereas MEC cases predominantly presented with stage I disease (43.9% vs 27.3%, *P* < .001). Both ACC and MEC patients had a high prevalence of T1 stage tumors (ACC: 29.0%, MEC: 46.6%; *P* < .001) and N0 status (ACC: 81.5%, MEC: 81.3%; *P* = .0118). Regarding treatment modalities, ACC patients were less likely to undergo surgery without parotidectomy (local tumor excision: 21.5% vs 9.9%) but more likely to receive a radical surgical approach (radical parotidectomy: 7.6% vs 4.1%, *P* < .001). Radiotherapy was more frequently administered to ACC patients compared to MEC patients (72.9% vs 43.8%, *P* < .001), whereas chemotherapy was used less commonly in ACC patients than in MEC patients (6.3% vs 10.4%, *P* < .001).

**Table 1:** Baseline characteristics of 1426 ACC and 3190 MEC patients from SEER.

	ACC (n = 1426)	MEC (n = 3190)	*P*
Age (yr)			
<10	1 (0.1%)	22 (0.7%)	<.001
10–19	11 (0.8%)	122 (3.8%)	
20–29	74 (5.2%)	251 (7.9%)	
30–39	141 (9.9%)	310 (9.7%)	
40–49	238 (16.7%)	483 (15.1%)	
50–59	320 (22.4%)	608 (19.1%)	
60–69	291 (20.4%)	647 (20.3%)	
70–79	234 (16.4%)	458 (14.4%)	
≥80	116 (8.1%)	289 (9.1%)	
Year			
2004	100 (7.0%)	180 (5.6%)	.2062
2005	73 (5.1%)	187 (5.9%)	
2006	96 (6.7%)	205 (6.4%)	
2007	89 (6.2%)	175 (5.5%)	
2008	89 (6.2%)	242 (7.6%)	
2009	73 (5.1%)	206 (6.5%)	
2010	109 (7.6%)	193 (6.1%)	
2011	97 (6.8%)	207 (6.5%)	
2012	92 (6.5%)	235 (7.4%)	
2013	95 (6.7%)	226 (7.1%)	
2014	90 (6.3%)	200 (6.3%)	
2015	101 (7.1%)	232 (7.3%)	
2018	106 (7.4%)	262 (8.2%)	
2019	113 (7.9%)	230 (7.2%)	
2020	103 (7.2%)	210 (6.6%)	
Race			
White	1094 (76.7%)	2418 (75.8%)	.0049
Black	140 (9.8%)	407 (12.8%)	
Other	192 (13.5%)	365 (11.4%)	
Sex			
Male	580 (40.7%)	1494 (46.8%)	<.001
Female	846 (59.3%)	1696 (53.2%)	
Primary site			
Parotid gland	666 (46.7%)	2758 (86.5%)	<.001
Submandibular gland	583 (40.9%)	289 (9.1%)	
Sublingual gland	61 (4.3%)	48 (1.5%)	
Overlapping lesions	4 (0.3%)	0 (0%)	
Other major salivary glands	112 (7.9%)	95 (3.0%)	
Surgery methods			
No surgery	136 (9.5%)	158 (5.0%)	<.001
Local tumor excision	306 (21.5%)	315 (9.9%)	
Less-than-total parotidectomy	404 (28.3%)	1489 (46.7%)	
Total parotidectomy	442 (31.0%)	1014 (31.8%)	
Radical parotidectomy	108 (7.6%)	131 (4.1%)	
Parotidectomy	14 (1.0%)	69 (2.2%)	
Surgery	16 (1.1%)	14 (0.4%)	
Radiotherapy			
Yes	1040 (72.9%)	1398 (43.8%)	<.001
No	386 (27.1%)	1792 (56.2%)	
Chemotherapy			
Yes	148 (10.4%)	201 (6.3%)	<.001
No	1278 (89.6%)	2989 (93.7%)	
Death			
Yes	522 (36.6%)	844 (26.5%)	<.001
No	904 (63.4%)	2346 (73.5%)	
Cause specific death			
Yes	357 (25.0%)	375 (11.8%)	<.001
No	1069 (75.0%)	2815 (88.2%)	
Stage			
I	389 (27.3%)	1401 (43.9%)	<.001
II	307 (21.5%)	673 (21.1%)	
III	285 (20.0%)	478 (15.0%)	
IV	445 (31.2%)	638 (20.0%)	
T			
TX/Tis	40 (2.8%)	33 (1.0%)	<.001
T0	7 (0.5%)	8 (0.3%)	
T1	413 (29.0%)	1486 (46.6%)	
T2	358 (25.1%)	813 (25.5%)	
T3	312 (21.9%)	459 (14.4%)	
T4	296 (20.8%)	391 (12.3%)	
N			
NX	23 (1.6%)	19 (0.6%)	.0118
N0	1162 (81.5%)	2593 (81.3%)	
N1	111 (7.8%)	262 (8.2%)	
N2	107 (7.5%)	271 (8.5%)	
N3	23 (1.6%)	45 (1.4%)	
M			
MX	2 (0.1%)	13 (0.4%)	<.001
M0	1271 (89.1%)	3097 (97.1%)	
M1	153 (10.7%)	80 (2.5%)	

ACC = adenoid cystic carcinoma, MEC = mucoepidermoid carcinoma, SEER = surveillance, epidemiology, and end results.

**Figure 2.**
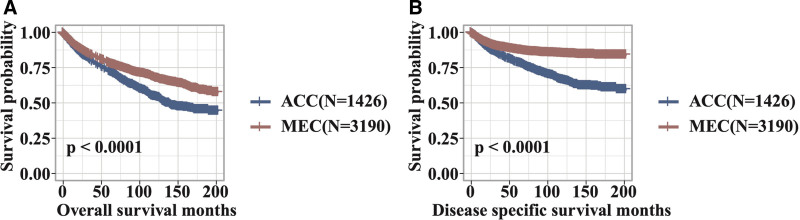
Kaplan–Meier analysis of overall survival (A) and disease-specific survival (B) for all ACC and MEC patients. ACC = adenoid cystic carcinoma, MEC = mucoepidermoid carcinoma.

Regarding survival outcomes, the overall mortality rate was significantly higher in the ACC group compared to the MEC group (36.6% vs 26.5%, *P* < .001), and ACC patients also had a higher rate of disease-specific mortality (25.0% vs 11.8%, *P* < .001). The mean OS for MEC was significantly higher than that for ACC (79.33 ± 58.62 months vs 73.44 ± 57.18 months, *P* < .001). Similarly, the mean DSS for MEC was significantly higher compared to ACC (46.80 ± 39.26 vs 29.06 ± 29.79 months, *P* < .001) (Fig. 2). Univariate and multivariate Cox regression analyses identified several independent predictors of OS in both ACC and MEC patients. For ACC, independent risk factors for OS included age ≥ 60 years (HR = 2.24, 95% CI = 1.86–2.69, *P* < .001), male gender (HR = 1.24, 95% CI = 1.04–1.48, *P* = .019), submandibular gland location (HR = 1.28, 95% CI = 1.05–1.56, *P* = .016), T3 stage (HR = 4.85, 95% CI = 1.13–20.85, *P* = .034), lymph node involvement (N1: HR = 1.81, 95% CI = 1.34–2.44, *P* < .001; N2: HR = 2.19, 95% CI = 1.60–3.00, *P* < .001; N3: HR = 4.61, 95% CI = 2.29–9.27, *P* < .001), and distant metastasis (HR = 2.31, 95% CI = 1.68–3.18, *P* < .001), while surgery (HR = 0.49, 95% CI = 0.36–0.67, *P* < .001) and radiotherapy (HR = 0.70, 95% CI = 0.57–0.85, *P* < .001) were protective factors. Independent risk factors for DSS included age ≥ 60 years (HR = 1.55, 95% CI = 1.24–1.93, *P* < .001), male gender (HR = 1.27, 95% CI = 1.02–1.58, *P* = .030), submandibular gland location (HR = 1.38, 95% CI = 1.08–1.77, *P* = .011), sublingual gland location (HR = 1.64, 95% CI = 1.05–2.58, *P* = .031), advanced stage (HR = 3.47, 95% CI = 1.31–9.19, *P* = .012), lymph node involvement (HRs ranging from 2.11–3.86, *P* < .001), and distant metastasis (HR = 2.20, 95% CI = 1.55–3.14, *P* < .001), while surgery was protective (HR = 0.40, 95% CI = 0.27–0.57, *P* < .001) (Tables 2 and 3).

**Table 2:** Univariate and multivariate analysis of overall survival for ACC and MEC patients.

	ACC				MEC			
	Univariate analysis		Multivariate analysis		Univariate analysis		Multivariate analysis	
	HR (95% CI for HR)	*P*	HR (95% CI for HR)	*P*	HR (95% CI for HR)	*P*	HR (95% CI for HR)	*P*
Age								
<60	1	<.001	1	<.001	1	<.001	1	<.001
≥60	2.32 (1.94–2.76)		2.24 (1.86–2.69)		5.06 (4.34–5.91)		4.33 (3.70–5.08)	
Sex								
Female	1	<.001	1	.019	1	<.001	1	.001
Male	1.41 (1.19–1.68)		1.24 (1.04–1.48)		1.90 (1.66–2.19)		1.27 (1.10–1.47)	
Race								
White	1	.40	–	–	1	<.001	1	.24
Black	1.06 (0.80–1.41)		–	–	0.78 (0.62–0.96)		0.87 (0.69–1.10)	
Other	0.85 (0.64–1.11)	–	–	–	0.43 (0.32–0.58)	–	0.51 (0.38–0.69)	<.001
Primary site								
Parotid gland	1	<.001	1	–	1	.04	1	–
Submandibular gland	1.07 (0.89–1.30)		1.28 (1.05–1.56)	.016	1.31 (1.05–1.62)		1.38 (1.10–1.72)	.0045
Sublingual gland	1.53 (1.03–2.26)		1.33 (0.89–1.99)	.16	0.63 (0.33–1.23)		0.72 (0.37–1.40)	.33
Other salivary glands	1.97 (1.47–2.66)		1.03 (0.73–1.46)	.86	1.15 (0.77–1.72)		0.83 (0.54–1.28)	.40
Overlapping lesions	0.98 (0.14–6.97)		1.22 (0.17–8.75)	.84	–		–	–
Surgery								
No	1	<.001	1	–	1	<.001	1	
Yes	0.20 (0.16–0.25)		0.49 (0.36–0.67)	<.001	0.13 (0.11–0.16)		0.34 (0.27–0.44)	<.001
Radiotherapy								
No	1	<.001	1		1	<.001		
Yes	0.71 (0.59–0.85)		0.70 (0.57–0.85)	<.001	1.73 (1.51–1.98)		0.94 (0.81–1.10)	.45
Chemotherapy								
No	1	<.001	1	<.001	1	<.001	1	.54
Yes	3.56 (2.82–4.49)		1.65 (1.27–2.16)		4.02 (3.33–4.86)		1.08 (0.85–1.36)	
Stage								
I	1	<.001	1		1	<.001	1	
II	1.89 (1.36–2.62)		1.35 (0.54–3.37)	.53	1.37 (1.09–1.72)		0.81 (0.49–1.36)	.43
III	2.56 (1.87–3.52)		0.99 (0.43–2.26)	.98	2.74 (2.23–3.37)		0.94 (0.58–1.51)	.79
IV	7.16 (5.44–9.43)		2.06 (0.87–4.88)	.10	6.05 (5.08–7.19)		1.60 (0.96–2.67)	.074
T								
T0	1	<.001	1	–	1	<.001	1	–
T1	0.36 (0.087–1.45)		1.95 (0.38–9.85)	.42	0.77 (0.11–5.48)		2.34 (0.31–17.73)	.41
T2	0.76 (0.19–3.09)		0.62 (12.14)	.19	1.31 (0.18–9.34)		4.07 (0.55–30.10)	.17
T3	1.21 (0.30–4.90)		4.85 (1.13–20.85)	.034	2.72 (0.38–19.43)		6.24 (0.85–45.80)	.072
T4	1.92 (0.48–7.75)		3.72 (0.88–15.76)	.074	3.89 (0.55–27.76)		5.02 (0.68–36.91)	.11
TX/Tis	6.15 (1.47–25.70)		1.85 (0.43–7.95)	.41	8.81 (1.19–65.14)		4.99 (0.66–37.85)	.12
N								
N0	1	<.001	1	–	1	<.001	1	–
N1	2.19 (1.66–2.89)		1.81 (1.34–2.44)	<.001	2.70 (2.22–3.29)		1.47 (1.14–1.89)	.0026
N2	4.90 (3.79–6.34)		2.19 (1.60–3.00)	<.001	4.78 (4.02–5.68)		1.61 (1.20–2.17)	.0016
N3	8.37 (4.26–16.44)		4.61 (2.29–9.27)	<.001	6.11 (3.86–9.69)		1.63 (0.96–2.77)	.072
NX	10.01 (6.56–15.30)		3.43 (2.09–5.62)	<.001	6.38 (3.75–10.85)		0.74 (0.38–1.47)	.39
M								
M0	1	<.001	1	<.001	1	<.001	1	<.001
M1	6.67 (5.39–8.25)		2.31 (1.68–3.18)		14.10 (11.02–18.05)		3.01 (2.17–4.17)	
MX	10.76 (1.50–76.94)		4.05 (0.55–29.92)	.17	3.31 (1.78–6.18)		0.83 (0.43–1.61)	.58

ACC = adenoid cystic carcinoma, CI = confidence interval, HR = hazard ratio, MEC = mucoepidermoid carcinoma.

**Table 3:** Univariate and multivariate analysis of disease-specific survival for ACC and MEC patients.

	ACC				MEC			
	Univariate analysis		Multivariate analysis		Univariate analysis		Multivariate analysis	
	HR (95% CI for HR)	*P*	HR (95% CI for HR)	*P*	HR (95% CI for HR)	*P*	HR (95% CI for HR)	*P*
Age								
<60	1	<.001	1	<.001	1	<.001	1	<.001
≥60	1.57 (1.27–1.93)		1.55 (1.24–1.93)		3.56 (2.86–4.44)		2.66 (2.12–3.34)	
Sex								
Female	1	.001	1	.030	1	<.001	1	.038
Male	1.40 (1.14–1.73)		1.27 (1.02–1.58)		2.44 (1.96–3.02)		1.27 (1.01–1.60)	
Race								
White	1	.50	–	–	1	<.001	1	.020
Black	1.19 (0.86–1.65)		–	–	0.63 (0.44–0.90)		0.64 (0.44–0.93)	
Other	0.92 (0.67–1.27)	–	–	–	0.41 (0.27–0.64)	–	0.51 (0.33–0.80)	.0033
Primary site								
Parotid gland	1	<.001	1	–	1	<.001	1	–
Submandibular gland	1.12 (0.89–1.41)		1.38 (1.08–1.77)	.011	1.55 (1.15–2.11)		1.53 (1.12–2.09)	.0073
Sublingual gland	1.86 (1.20–2.89)		1.64 (1.05–2.58)	.031	0.52 (0.17–1.62)		0.73 (0.23–0.45)	.60
Other salivary glands	2.00 (1.40–2.87)		0.92 (0.60–1.41)	.70	2.08 (1.31–3.31)		1.38 (0.81–2.36)	.24
Overlapping lesions	1.39 (0.19–9.93)		1.59 (0.22–11.47)	.64	–		–	–
Surgery								
No	1	<.001	1	–	1	<.001	1	
Yes	0.17 (0.13–0.22)		0.40 (0.27–0.57)	<.001	0.091 (0.071–0.12)		0.33 (0.24–0.45)	<.001
Radiotherapy								
No	1	0.2	–	–	1	<.001	1	–
Yes	0.87 (0.69–1.10)		–	–	3.30 (2.64–4.13)		1.21 (0.94–1.56)	.14
Chemotherapy								
No	1	<.001	1		1	<.001	1	
Yes	4.71 (3.65–6.08)		1.76 (1.31–2.36)	<.001	6.97 (5.52–8.79)		1.09 (0.82–1.44)	.56
Stage								
I	1	<.001	1		1	<.001	1	–
II	2.43 (1.51–3.90)		1.68 (0.59–4.74)	.33	1.75 (1.04–2.96)		1.26 (0.58–2.74)	.56
III	3.65 (2.32–5.75)		1.40 (0.55–3.56)	.48	7.97 (5.29–12.00)		2.37 (1.22–4.61)	.011
IV	12.93 (8.65–19.31)		3.47 (1.31–9.19)	.012	22.66 (15.65–32.79)		4.38 (2.15–8.89)	<.001
T								
T0	1	<.001	1		1	<.001	1	
T1	0.36 (0.049–2.63)		2.79 (0.32–24.56)	0.35	0.17 (0.024–1.27)		2.53 (0.32–20.18)	0.38
T2	1.03 (0.14–7.39)		3.87 (0.49–30.38)	0.20	0.45 (0.063–3.26)		3.43 (0.45–26.41)	0.34
T3	1.72 (0.24–12.31)		6.29 (0.82–48.22)	0.077	1.60 (0.22–11.45)		6.21 (0.82–47.19)	0.078
T4	3.10 (0.43–22.20)		4.71 (0.63–35.31)	0.13	2.18 (0.30–15.60)		4.44 (0.58–33.83)	0.15
TX/Tis	10.22 (1.39–75.36)		2.97 (0.39–22.42)	0.29	6.99 (0.94–51.97)		3.58 (0.46–27.69)	0.22
N								
N0	1	<.001	1		1	<.001	1	
N1	3.00 (2.20–4.09)		2.11 (1.50–2.96)	<.001	6.00 (4.56–7.90)		2.00 (1.44–2.79)	<.001
N2	7.29 (5.50–9.66)		2.53 (1.79–3.56)	<.001	11.48 (9.03–14.60)		2.24 (1.53–3.28)	<.001
N3	10.34 (4.79–22.29)		3.86 (1.76–8.49)	<.001	13.70 (8.02–23.42)		2.01 (1.08–3.74)	.028
NX	15.24 (9.71–23.91)		3.90 (2.29–6.63)	<.001	17.64 (9.56–32.58)		1.13 (0.51–2.51)	.76
M								
M0	1	<.001	1		1	<.001	1	
M1	8.22 (6.45–10.47)		2.20 (1.55–3.14)	<.001	22.62 (17.07–29.96)		3.59 (2.46–5.22)	<.001
MX	15.15 (2.11–108.67)		3.41 (0.46–25.46)	.23	4.03 (1.67–9.76)		0.76 (0.30–1.91)	.56

ACC = adenoid cystic carcinoma, CI = confidence interval, HR = hazard ratio, MEC = mucoepidermoid carcinoma.

For MEC, independent risk factors for OS were age ≥ 60 years (HR = 4.33, 95% CI = 3.70–5.08, *P* < .001), male gender (HR = 1.27, 95% CI = 1.10–1.47, *P* = .001), other race (HR = 0.51, 95% CI = 0.38–0.69, *P* < .001), submandibular gland location (HR = 1.38, 95% CI = 1.10–1.72, *P* = .0045), lymph node involvement (N1: HR = 1.47, 95% CI = 1.14–1.89, *P* = .0026; N2: HR = 1.61, 95% CI = 1.20–2.17, *P* = .0016), and distant metastasis (HR = 3.01, 95% CI = 2.17–4.17, *P* < .001), while surgery was associated with improved survival (HR = 0.34, 95% CI = 0.27–0.44, *P* < .001). Independent risk factors for DSS were age ≥ 60 years (HR = 2.66, 95% CI = 2.12–3.34, *P* < .001), male gender (HR = 1.27, 95% CI = 1.01–1.60, *P* = .038), nonwhite race (HR = 0.51, 95% CI = 0.33–0.80, *P* = .0033), submandibular gland location (HR = 1.53, 95% CI = 1.12–2.09, *P* = .0073), advanced stage (HRs for stage III and IV ranging from 2.37–4.38, *P* ≤ .011), lymph node involvement (HRs ranging from 2.00–2.24, *P* < .001), and distant metastasis (HR = 3.59, 95% CI = 2.46–5.22, *P* < .001), with surgery being a protective factor (HR = 0.33, 95% CI = 0.24–0.45, *P* < .001) (Tables 2 and 3).

## 4. Discussion

This study showed that MEC had significantly better OS and DSS compared to ACC. Age ≥60 years, male gender, submandibular gland involvement, advanced nodal status, and distant metastasis were associated with poorer outcomes for both cancer types, while surgery was a critical protective factor.

In this study, we found no significant difference in the number of MEC and ACC cases between 2004 and 2020. However, the total number of MEC cases was more than double that of ACC in the SEER database. The peak onset age for ACC was younger than for MEC, and ACC patients had a higher proportion of advanced disease, suggesting a worse prognosis for ACC. Due to the data source, the majority of cases were observed in Caucasians. The impact of sex on clinical outcomes in head and neck ACC remains controversial. We found that more women were diagnosed with both ACC and MEC, and they had a better prognosis compared to men, consistent with previous large epidemiological studies.^[5,13]^ Ellington et al suggested that sex-specific survival differences might be due to hormonal influences or better adherence to treatment in women.^[2]^ However, another study reported that female sex was linked to a higher rate of ACC recurrence.^[14]^

In terms of tumor location, ACC was more frequently observed in the parotid gland, followed by the submandibular gland, while MEC occurred predominantly in the parotid gland. Prognosis was worse for tumors in the submandibular gland compared to the parotid gland in both ACC and MEC, aligning with previous findings.^[15,16]^ The common surgical procedures for ACC and MEC included total and less-than-total parotidectomy, with ACC patients undergoing local tumor excision more often.^[12]^ The OS times following radical and total parotidectomy were similar between ACC and MEC, but for other surgical approaches, OS was shorter for ACC compared to MEC.

Radiotherapy was used more frequently in ACC than in MEC, and it significantly improved prognosis for ACC, while it had no significant impact on MEC outcomes.^[10,17]^ Chemotherapy had a negative effect on ACC prognosis and did not show clear benefits for MEC, in line with previous studies indicating limited efficacy of chemotherapy for MEC and potential detrimental effects on OS.^[18]^ Ferrell et al also reported that surgery combined with chemoradiation worsened OS compared to surgery alone in salivary gland cancers.^[19]^ ACC patients showed an even distribution across stages I to IV, whereas MEC patients were more frequently diagnosed in early stages (I and T1), indicating that ACC is often detected later due to a lack of early clinical symptoms and the frequent presence of neurological invasion at the time of diagnosis. Interestingly, the proportion of ACC patients with distant metastasis was 10%, which is lower than previous reports (25%–55%),^[20]^ while only 2.5% of MEC patients had distant metastasis. Among patients with distant metastasis, ACC showed significantly longer OS and DSS compared to MEC, suggesting that ACC patients with distant metastasis may have a relatively longer survival period. However, overall, ACC had significantly shorter OS than MEC, indicating poorer prognosis for ACC. Unlike the complexity of radiotherapy and chemotherapy outcomes, surgery was a significant independent factor for improving prognosis in both ACC and MEC.^[21–23]^ ACC’s aggressive nature contributes to its poorer prognosis, highlighting the need for effective adjuvant therapies. MEC generally showed better outcomes, especially in early-stage cases. These findings suggest that individualized treatment, particularly aggressive management for high-risk patients, is crucial. Surgical intervention remains a key strategy for both cancers. Future research should explore novel therapies and multimodal approaches, especially for ACC, to improve patient survival.

This study has several limitations that should be acknowledged. First, being a retrospective analysis, this study is subject to inherent biases related to data collection and patient selection. Differences in baseline characteristics between the ACC and MEC groups may have led to selection bias, potentially impacting the reliability of the comparative results. Second, the smaller sample size of ACC patients compared to MEC, especially in those receiving chemotherapy, may have influenced the statistical power and accuracy of the conclusions. Third, the SEER database, while comprehensive, lacks critical information on chemotherapy regimens, immunotherapy, targeted therapies, and recurrence status – factors that are highly relevant to understanding patient prognosis and treatment efficacy. The absence of these data limited our ability to perform more detailed analyses of treatment effects and disease progression, which could have provided a more nuanced understanding of survival outcomes for both ACC and MEC patients. Additionally, factors such as treatment adherence, comorbidities, and patient quality of life, which could significantly impact prognosis, were not available in the dataset, limiting the depth of clinical insights that could be derived from this study. Future research should focus on prospective studies with a more comprehensive data collection framework, including detailed treatment protocols and patient outcomes, to address these limitations and provide more robust guidance for clinical practice.

## 5. Conclusion

In conclusion, this study demonstrated that MEC had significantly better OS and DSS compared to ACC. Poorer outcomes in both cancer types were associated with age ≥60 years, male gender, submandibular gland involvement, advanced nodal status, and distant metastasis, whereas surgical intervention significantly improved survival. These results highlight the necessity of early diagnosis and individualized treatment approaches, particularly for high-risk patients. Future research should focus on gathering more comprehensive data to refine management strategies for these malignancies.

## Author contributions

**Data curation:** Xiaoqin Yang, Ye Meng, Can Xiao, Liya Wang.

**Formal analysis:** Xiaoqin Yang, Ye Meng, Can Xiao, Liya Wang.

## References

[1] Fang Y Peng Z Wang Y. Current opinions on diagnosis and treatment of adenoid cystic carcinoma. Oral Oncol. 2022;130:105945. 35662026 10.1016/j.oraloncology.2022.105945

[2] Ellington CL Goodman M Kono SA. Adenoid cystic carcinoma of the head and neck: incidence and survival trends based on 1973‐2007 surveillance, epidemiology, and end results data. Cancer. 2012;118:4444–51. 22294420 10.1002/cncr.27408

[3] Peraza A Gómez R Beltran J Amarista F. Mucoepidermoid carcinoma. An update and review of the literature. J Stomatol Oral Maxillofac Surg. 2020;121:713–20. 32565266 10.1016/j.jormas.2020.06.003

[4] Jaso J Malhotra R. Adenoid cystic carcinoma. Arch Pathol Lab Med. 2011;135:511–5. 21466371 10.5858/2009-0527-RS.1

[5] Coca-Pelaz A Rodrigo JP Bradley PJ. Adenoid cystic carcinoma of the head and neck – an update. Oral Oncol. 2015;51:652–61. 25943783 10.1016/j.oraloncology.2015.04.005

[6] Geiger JL Ismaila N Beadle B. Management of salivary gland malignancy: ASCO guideline. J Clin Oncol. 2021;39:1909–41. 33900808 10.1200/JCO.21.00449

[7] Sama S Komiya T Guddati AK. Advances in the treatment of mucoepidermoid carcinoma. World J Oncol. 2022;13:1–7. 35317327 10.14740/wjon1412 PMC8913015

[8] Sun M Qu Y Wang K. Long-term outcomes of patients in different histological subtypes of primary nasopharyngeal adenocarcinoma: a single-center experience with 71 cases. Oral Oncol. 2020;111:104923. 32795912 10.1016/j.oraloncology.2020.104923

[9] Byrd SA Spector ME Carey TE Bradford CR McHugh JB. Predictors of recurrence and survival for head and neck mucoepidermoid carcinoma. Otolaryngol Head Neck Surg. 2013;149:402–8. 23695589 10.1177/0194599813489659 PMC4106041

[10] Orlandi E Sanguineti G Fallai C. Salivary gland tumors: radiotherapy. In: Licitra L Locati L, eds. Salivary Gland Cancer: From Diagnosis to Tailored Treatment. Springer; 2019:159–93.

[11] Rauh-Hain JA Vargas RJ Clemmer J. Mucinous adenocarcinoma of the endometrium compared with endometrioid endometrial cancer: a SEER analysis. Am J Clin Oncol. 2016;39:43–8. 24390270 10.1097/COC.0000000000000015

[12] Vander Poorten V. Surgery for malignant parotid gland tumours. In: Licitra L Locati L, eds. Salivary Gland Cancer: From Diagnosis to Tailored Treatment. Springer; 2019:45–67.

[13] Ciccolallo L Licitra L Cantú G Gatta G; EUROCARE Working Group. Survival from salivary glands adenoid cystic carcinoma in European populations. Oral Oncol. 2009;45:669–74. 19095489 10.1016/j.oraloncology.2008.10.010

[14] Marcinow A Ozer E Teknos T. Clinicopathologic predictors of recurrence and overall survival in adenoid cystic carcinoma of the head and neck: a single institutional experience at a tertiary care center. Head Neck. 2014;36:1705–11. 24166847 10.1002/hed.23523 PMC4299584

[15] Bosch A Brandenburg JH Gilchrist KW. Lymph node metastases in adenoid cystic carcinoma of the submaxillary gland. Cancer. 1980;45:2872–7. 6247058 10.1002/1097-0142(19800601)45:11<2872::aid-cncr2820451125>3.0.co;2-0

[16] Thomson DJ Slevin NJ Mendenhall WM. Indications for salivary gland radiotherapy. Adv Oto-Rhino-Laryngol. 2016;78:141–7. 10.1159/000442134 27093301

[17] de Souza LB de Oliveira LC Nonaka CFW Lopes MLDS Pinto LP Queiroz LMG. Immunoexpression of GLUT-1 and angiogenic index in pleomorphic adenomas, adenoid cystic carcinomas, and mucoepidermoid carcinomas of the salivary glands. Eur Arch Otorhinolaryngol. 2017;274:2549–56. 28299426 10.1007/s00405-017-4530-y

[18] Rajasekaran K Stubbs V Chen J. Mucoepidermoid carcinoma of the parotid gland: a National Cancer Database study. Am J Otolaryngol. 2018;39:321–6. 29559174 10.1016/j.amjoto.2018.03.022

[19] Ferrell JK Mace JC Clayburgh D. Contemporary treatment patterns and outcomes of salivary gland carcinoma: a National Cancer Database review. Acta Otorhinolaryngol Ital. 2019;276:1135–46. 10.1007/s00405-019-05282-2 30649610

[20] Dillon PM Chakraborty S Moskaluk CA Joshi PJ Thomas CY. Adenoid cystic carcinoma: a review of recent advances, molecular targets, and clinical trials. Head Neck. 2016;38:620–7. 25487882 10.1002/hed.23925 PMC6166139

[21] Cantù G. Adenoid cystic carcinoma. An indolent but aggressive tumour. Part B: treatment and prognosis. Acta Otorhinolaryngol Ital. 2021;41:296–307. 34533533 10.14639/0392-100X-N1729 PMC8448184

[22] Chan SA Van Abel KM Lewis JE. Mucoepidermoid carcinoma of the parotid gland: twenty‐year experience in treatment and outcomes. Head Neck. 2021;43:2663–71. 33931913 10.1002/hed.26735

[23] Chen MM Roman SA Sosa JA Judson BL. Histologic grade as prognostic indicator for mucoepidermoid carcinoma: a population‐level analysis of 2400 patients. Head Neck. 2014;36:158–63. 23765800 10.1002/hed.23256

